# Squamous cell carcinoma around a subperiosteal implant in the maxilla and the association of chronic mechanical irritation and peri-implantitis: a case report

**DOI:** 10.1186/s40729-022-00409-3

**Published:** 2022-03-02

**Authors:** Takuma Watanabe, Dai Kawahara, Ryo Inoue, Tomoki Kato, Noboru Ishihara, Hidemichi Kamiya, Kazuhisa Bessho

**Affiliations:** 1 Department of Oral and Maxillofacial Surgery, Graduate School of Medicine, Kyoto University, 54 Kawahara-cho, Shogoin, Sakyo-ku, Kyoto, 606-8507 Japan; 2 Institute of Clinical Materials, 1-22-27 Tokocho, Moriguchi, Osaka 570-0035 Japan

**Keywords:** Subperiosteal implants, Chronic mechanical irritation, Peri-implantitis, Squamous cell carcinoma, Persistent inflammation

## Abstract

**Background:**

As carcinogenic risk factors, environmental factors can be classified into physical, biological, and chemical factors. Subperiosteal implants (SIs) are associated with complications, such as framework exposure, infection, and fistula formation. A current hypothesis suggests that chronic mechanical irritation could be a co-factor in carcinogenesis, while peri-implantitis might be an initiating or promoting agent in the development of oral mucosal cancer. Herein, we report a case of squamous cell carcinoma (SCC) around a maxillary SI associated with chronic mechanical irritation and peri-implantitis as physical and biological factors, respectively.

**Case presentation:**

A 74-year-old male patient presented with severe mobility of the SI and an undermined ulcer with induration, accompanied by a palatal fistula and the exposure of the metal framework. The SI had been placed on the maxilla for the occlusal reconstruction of the molar area 20 years ago. An incisional biopsy of the ulcer revealed SCC (cT4aN2cM0). Neoadjuvant chemotherapy was initiated, followed by bilateral neck dissection and partial resection of the maxilla with SI removal. Energy-dispersive X-ray analysis suggested that the SI was fabricated using pure titanium, and titanium was absent in the specimen. Scanning electron microscopy of the SI in contact with the SCC showed a few microcracks, suggesting pitting corrosion.

**Discussion:**

Chronic mechanical irritation due to the mobility of an improperly designed SI can be a physical factor, and prolonged peri-implantitis without regular maintenance can be a biological factor in carcinogenesis. Improperly designed main struts and a large masticatory force in the molar area resulted in deterioration of the retention and mobility of the SI. The screw and framework frequently moved on mastication and came in direct contact with the ulcer as chronic mechanical irritation. Bacterial invasion into the subperiosteal space expanded by the mobility of the metal framework led to peri-implantitis. The influence of chemical factors was considered relatively small in this case since the patient had no history of smoking or drinking, and titanium was absent in the specimen. Therefore, it is conceivable that SCC can arise owing to persistent inflammation caused by chronic mechanical irritation and peri-implantitis as physical and biological factors, respectively.

## Background

As carcinogenic risk factors, environmental factors can be representatively classified into physical, biological, and chemical factors [1, 2]. The physical factors include ionizing radiation, ultraviolet, or mechanical irritation, while the biological factors include various viruses or microbes [1–6]. The chemical factors mainly include tobacco or alcohol [1, 2, 6].

Subperiosteal implants (SIs) were first introduced in the 1940s and have been used as a conservative option for implant treatment of severely resorbed maxilla and mandible since then [7–9]. An SI may be a viable treatment option in patients with inadequate bone volume for endosseous implants. SIs reduce the treatment time required to restore lost prosthetic function and, possibly, lower the financial burden for some patients [7, 10]. Conversely, several types of complications, including pain, primary exposure of the implant framework, inflammation, infection, fistula formation, late implant exposure, bone resorption, and implant mobility, have been associated with the use of SIs [11].

Chronic mechanical irritation, which is generated through low-intensity, sustained, and repeated action of a deleterious agent in the oral cavity, can be caused by teeth, dentures, and functional alterations [4]. Peri-implantitis, a condition characterized by inflammation and loss of the supporting tissue around dental implants, rarely results in inflammation-induced carcinogenesis [12, 13]. A hypothesis suggests that chronic mechanical irritation could act as a co-factor in carcinogenesis [5, 6], while peri-implantitis might act as an initiating or promoting agent in the development of mucosal cancer [13].

Although the development of squamous cell carcinoma (SCC) in the vicinity of dental implants is a rare event, case reports of oral malignancies associated with dental implants have gradually accumulated [12, 14, 15]. However, to the best of our knowledge, there are no reports of SCC occurring around an SI. This report presents a rare case of SCC around a maxillary SI associated with chronic mechanical irritation and peri-implantitis as physical and biological factors, respectively.

## Case presentation

A 74-year-old male patient was referred to our department with severe pain in the palate and nasal obstruction. His medical history included hypertension and type 2 diabetes mellitus, for which he was taking azilsartan and metformin hydrochloride, respectively. He had no history of smoking or drinking. In 1999, a maxillary SI, on which the metal framework was designed to be fixed with two retaining screws, was placed on a severely atrophied maxilla for the occlusal reconstruction of the molar area. One of the retaining screws fell off shortly after the implant placement. Although he had used the SI without any particular dysfunction during mastication during the 5-year follow-up, he had experienced mild pain in the palate and slight mobility of the SI. After the follow-up ended, the mobility of the SI gradually worsened. Subsequently, he developed nasal obstruction in 2017, followed by severe pain in the palate. He later visited our department in 2019.

**Fig. 1:**
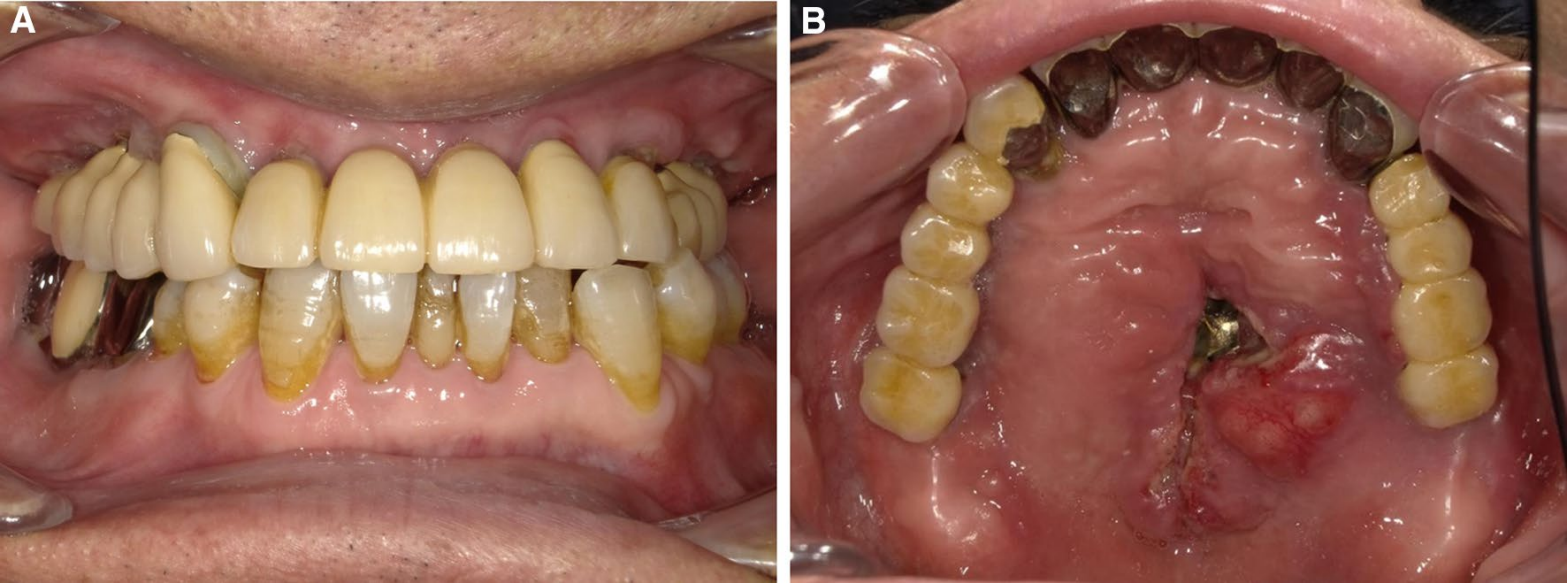
Frontal view of an intraoral photograph showing a molar prosthesis attached to the metal framework (**A**). Palatal view of an intraoral photograph showing the metal framework exposed through the fistula with an undermined ulcer (**B**)

**Fig. 2:**
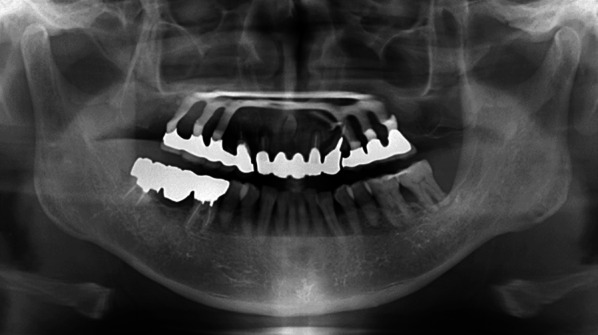
Panoramic radiograph showing a maxillary subperiosteal implant and retaining screw

**Fig. 3:**
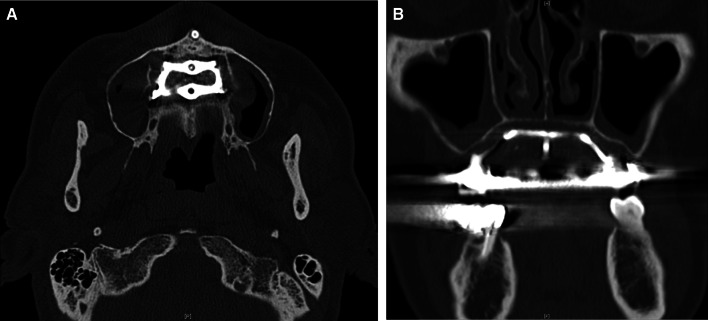
Axial (**A**) and coronal (**B**) computed tomography images showing major maxillary bone resorption around the metal framework, the retaining screw that almost fell off, and bilateral maxillary sinusitis

**Fig. 4:**
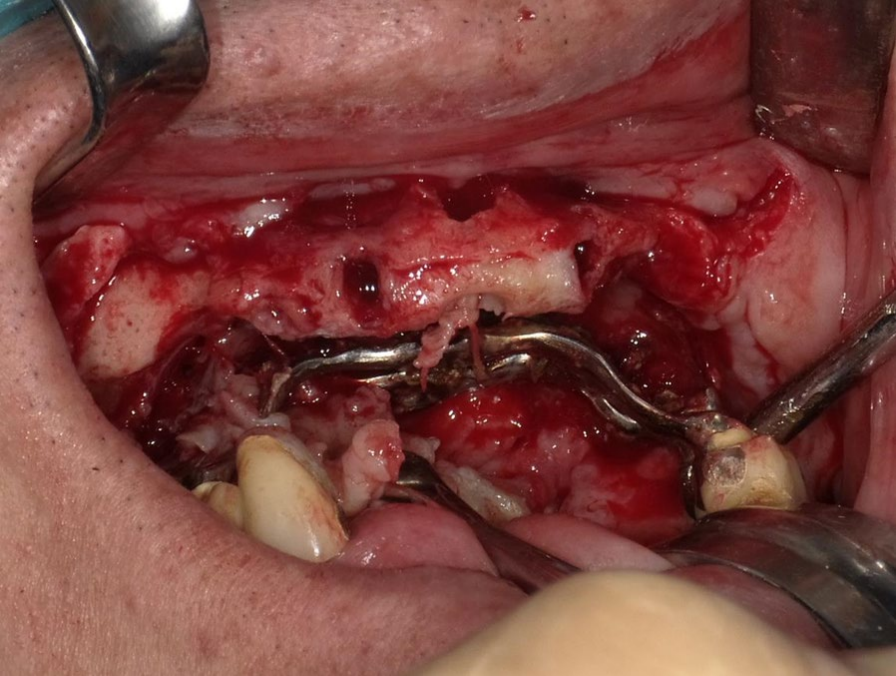
Intraoperative photograph showing the subperiosteal implant being removed

**Fig. 5:**
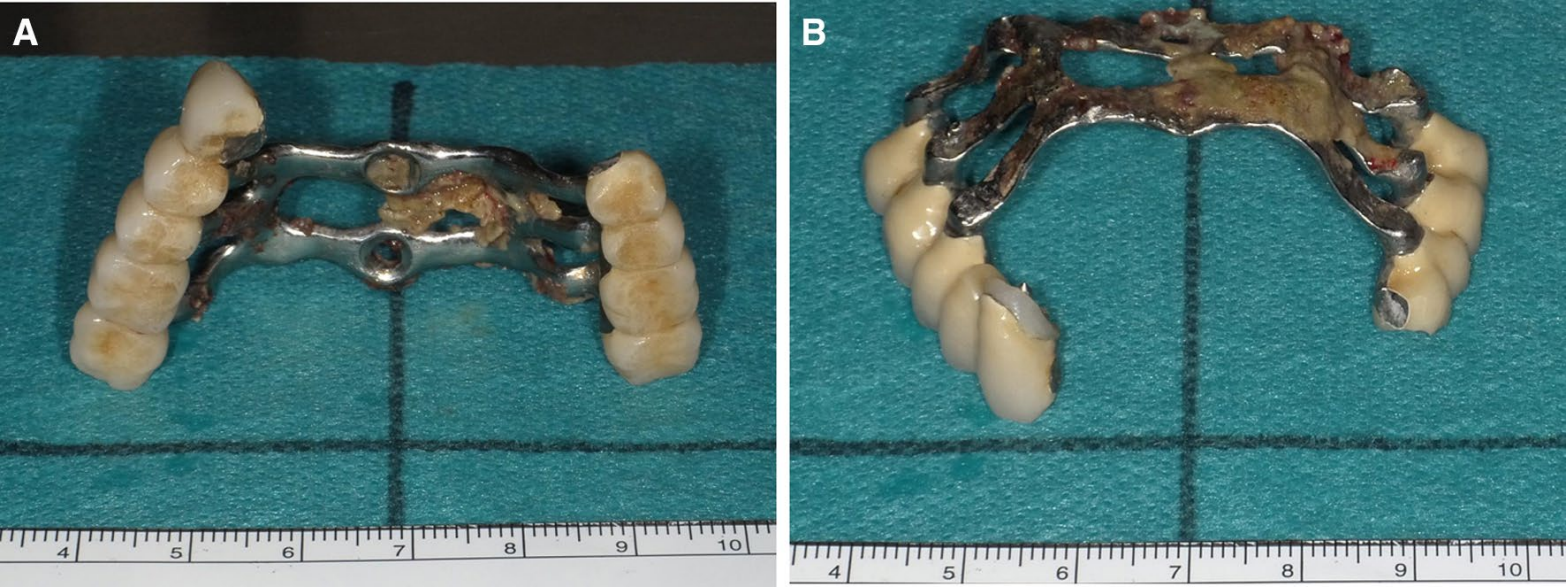
Subperiosteal implant comprising the metal framework and attachments of the prosthesis; the surface is covered with plaque and calculus (**A**, **B**)

**Fig. 6:**
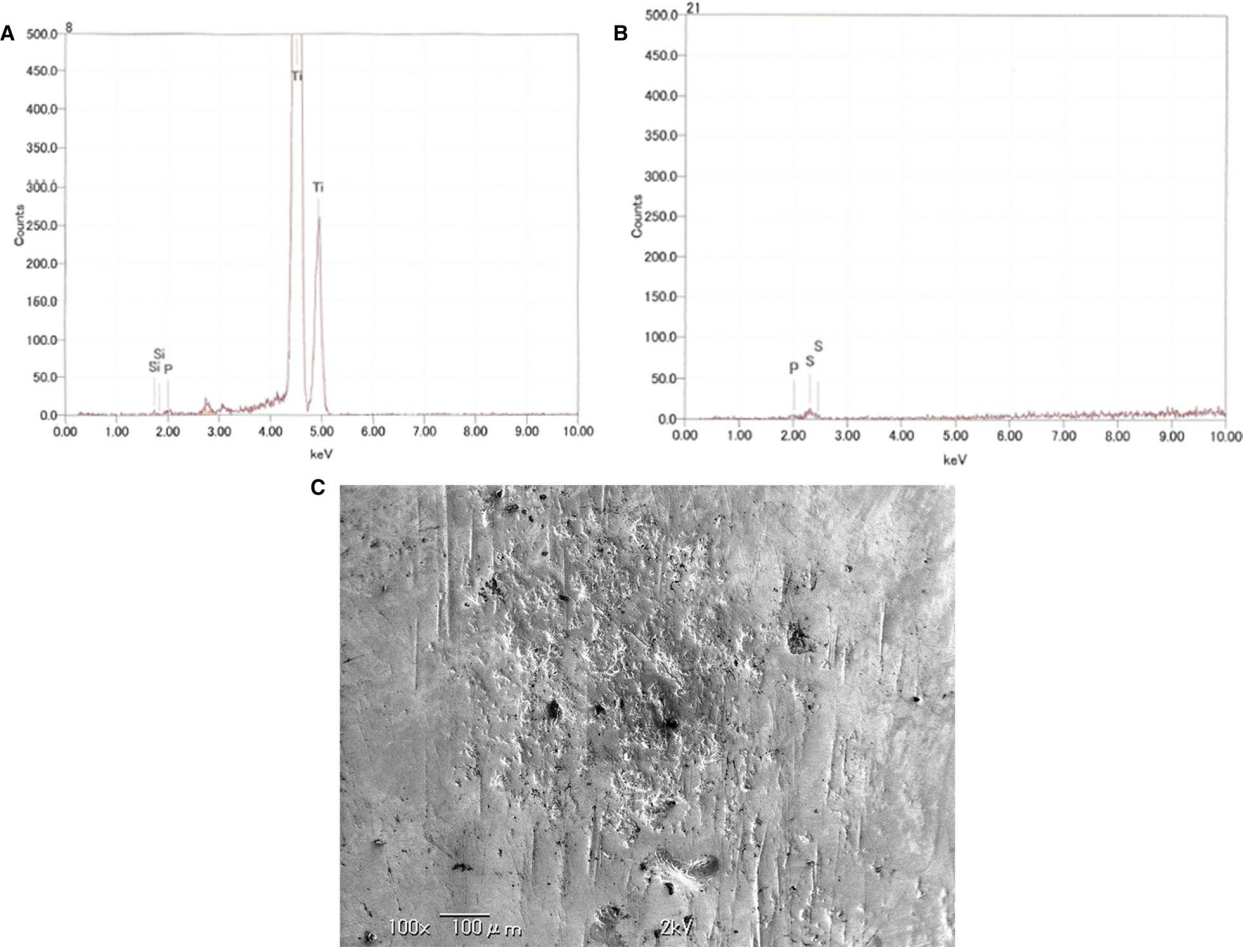
Energy-dispersive X-ray analysis (EDX) of the subperiosteal implant (SI) shows a few elements (Ti, Si, and P) (**A**), while EDX of the resected specimen shows Si and P, but not titanium (**B**). Scanning electron microscopy of the SI in contact with the squamous cell carcinoma shows a few microcracks (**C**)

**Fig. 7:**
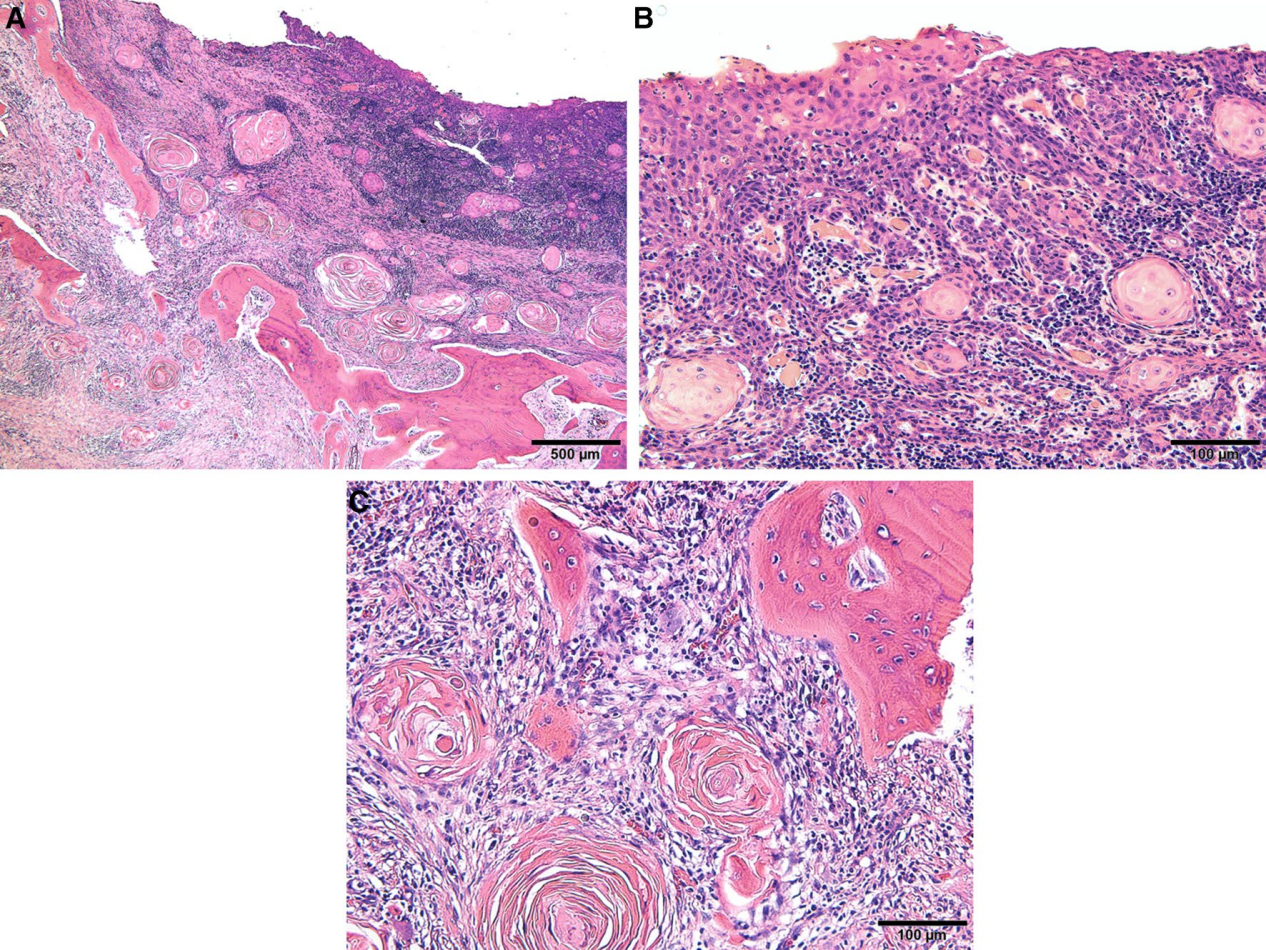
Histopathologic examination following hematoxylin–eosin staining shows well-differentiated squamous cell carcinoma, which was highly keratinized (**A**). The epithelium shows widespread ulceration and extensive infiltration of the stroma (**B**) and bone (**C**)

**Fig. 8:**
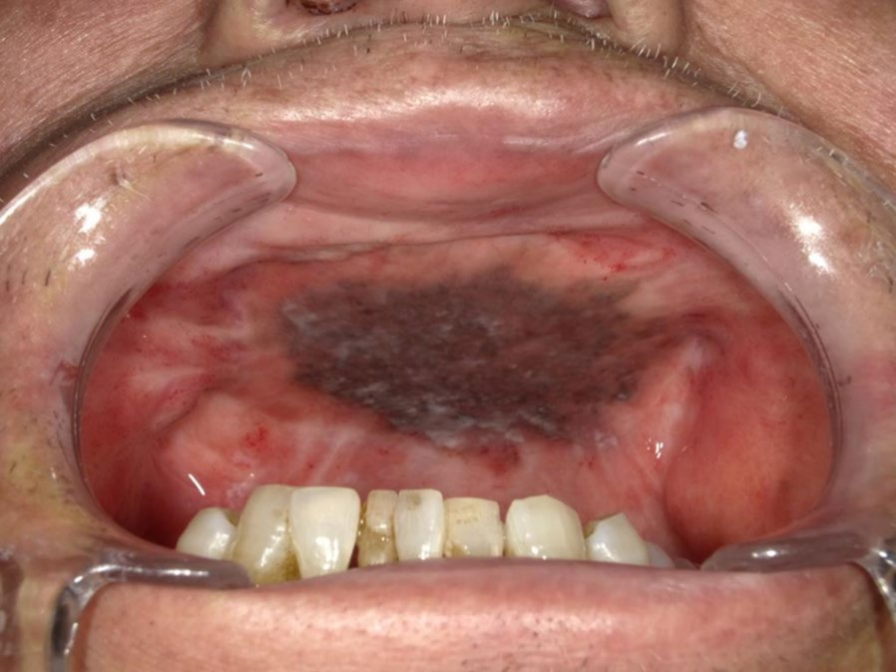
Intraoral photograph showing the reconstructed palate with the loss of the alveolar ridge

Clinical examination revealed a maxillary SI with poor fitting, severe mobility, and an undermined ulcer with induration on the palate, accompanied by a fistula and the exposure of the metal framework (Fig. 1A, B). A panoramic radiograph showed a maxillary SI and retaining screw (Fig. 2), while computed tomography showed major maxillary bone resorption around the metal framework, the retaining screw that almost fell off, and bilateral maxillary sinusitis (Fig. 3A, B). An incisional biopsy was performed under the suspicion of carcinoma, and pathological and systemic examination revealed SCC (cT4aN2cM0). After administering neoadjuvant chemotherapy comprising paclitaxel, carboplatin, and cetuximab, bilateral neck dissection was performed, followed by partial resection of the maxilla with the removal of the SI (Fig. 4). The surgical defect of the palate was reconstructed with a free anterolateral thigh flap. The SI, which was removed, comprised a metal framework with two holes for retaining screws and attachments of the prosthesis of the molar region. The surface in contact with the bone was covered with extensive amounts of plaque and calculus, while the polished surface in contact with the mucoperiosteum was covered with a small amount of calculus (Fig. 5A, B). Energy-dispersive X-ray (EDX) analysis suggested that the SI was fabricated using pure titanium on a cast (Fig. 6A) and that no titanium was present in the resected specimen (Fig. 6B). Scanning electron microscopy (SEM) of the SI in contact with the SCC showed a few microcracks, suggesting pitting corrosion (Fig. 6C). Pathological microscopic examination of the resected primary tumor revealed well-differentiated SCC, which was highly keratinized. It showed widespread ulceration of the epithelium and extensive infiltration of the stroma and bone (Fig. 7A–C). Subsequently, he underwent adjuvant chemoradiotherapy, which comprised cisplatin combined with intensity-modulated radiation therapy, as the resection margins were positive. Wound healing of the reconstructed palate and dietary intake were uneventful (Fig. 8). Regarding the prosthetic solution, it was difficult to provide a maxillary denture with sufficient stability due to the loss of the alveolar ridge. Currently, he is undergoing chemotherapy for recurrent lymph node metastasis.

## Discussion

The patient’s course suggested important clinical issues. Although a well-designed SI can function successfully for many years, chronic mechanical irritation caused by the mobility of an improperly designed SI can be a physical factor in carcinogenesis. More importantly, prolonged peri-implantitis without regular maintenance can act as a biological factor in carcinogenesis, although tobacco and alcohol are often considered the major risk factors for oral cancer.

SIs fitting the shape of the jaw are integrated into the subperiosteal layer of the alveolus and held in place by a fibrous connection generated by the overlying periosteum [16]. A properly designed SI can function successfully for a significant period [17], with bone morphology and texture playing major roles in determining its success [18]. In the case of a maxillary SI, the anterior nasal spine, zygomatic buttresses, canine pillars, and tuberosities are the main struts of the SI for stability against vertical and lateral forces [18]. Previous studies have suggested that the higher failure rate of maxillary SIs than mandibular SIs could be due to the following: (1) the negative effect of gravity on the maintenance of a good bone–implant contact zone and (2) difficulty in positioning maxillary implants on a cortical bone basis [8, 17]. Furthermore, the use of SIs is contraindicated when the opposing teeth are natural [16]. In this case, the main struts of the SI were not designed to accommodate anatomic landmarks, and the bone–implant contact area was small. In addition, one of the two retaining screws fell off immediately, and the opposing teeth had a lower molar with a large masticatory force. These factors resulted in the deterioration of the retention and mobility of the SI.

Available evidence indicates that chronic mechanical irritation could act as a tumor promoter [5]. Recurrent persistent inflammation due to chronic mechanical irritation may induce or promote carcinogenesis by DNA damage, inciting cell proliferation, and the release of cytokines and growth factors [5, 19]. Further, chronic mechanical irritation can be considered a factor affecting carcinogenesis when all of the following conditions are registered: (1) objective clinical cancerous lesions compatible with a mechanical origin; (2) mechanical factors present before the onset of the cancerous lesion, and (3) mechanical agent in direct contact with the cancerous lesion during functional/parafunctional movements [3, 4]. In this case, the remaining screw and metal framework with prolonged mobility were present before the onset of the SCC. The screw and framework placed on the maxilla frequently moved during mastication and were in direct contact with the palatal tissue after the formation of the fistula.

The characteristics of peri-implantitis, which is the most common peri-implant lesion, include swelling, erythema, suppuration, and bone loss [20]. In addition, chronic infections associated with a failing SI can manifest as extraoral/intraoral fistulas [16]. Various mechanisms underlying carcinogenesis related to persistent inflammation, which is regarded as a possible risk factor, have been suggested [15]. The concept of inflammation-induced cancer was explored with respect to free radicals, such as reactive oxygen and nitrogen species, generated by the inflammatory cells, which are suspected to play a pivotal role in causing mutations and carcinogenesis [12, 21]. In this case, mucous perforations around the six struts were a direct gateway for organisms from the oral cavity, and the organisms invaded the subperiosteal space expanded by the mobility of the metal framework. Eventually, the peri-implantitis accompanying the fistula occurred around the remaining loose screw.

Previously hypothesized factors regarding carcinogenesis associated with dental implant include the following: (1) dental implant corrosion; (2) the possible association between corrosion products and cancer; (3) the possible association between particulate titanium and cancer, and (4) the hypothesized carcinogenic effect of sustained metallic ion release after implant placement [22]. Conversely, titanium is one of the most inert metallic ions that is especially resistant to corrosion (with a corrosion rate of 0.003 µA/cm^2^) owing to the stability of the layer of titanium dioxide; however, inflammatory reactions, such as peri-implantitis, could upset the layer favoring possible corrosion [22–24]. In this case, EDX analysis showed no titanium in the resected specimen; conversely, SEM showed a few microcracks, suggesting pitting corrosion. In addition, the patient had no history of smoking or drinking; therefore, the influence of chemical factors on carcinogenesis was considered relatively small.

Ultimately, based on these clinical courses, it is conceivable that SCC can emerge through persistent inflammation caused by chronic mechanical irritation and peri-implantitis as physical and biological factors, respectively. Therefore, this case indicates that lesions associated with an exposed SI should be considered for possible malignancy.

## Data Availability

Not applicable.

## Abbreviations

SI: Subperiosteal implant
SCC: Squamous cell carcinoma
EDX: Energy dispersive X-ray
SEM: Scanning electron microscopy
